# Case report and literature review: T2DM with DKA, HHS and rhabdomyolysis

**DOI:** 10.1186/1687-9856-2013-S1-P19

**Published:** 2013-10-03

**Authors:** Wenjing Li, Chunxiux Gong, Di Wu, Bing Sun

**Affiliations:** 1 Pediatrics Endocrinology Department, Beijing Children's Hospital, China

## 

We report two type 2 diabetes mellitus (T2DM) adolescent patients who had diabetic ketoacidosis (DKA) and hyperglycemic hyperosmolar state (HHS) predisposing rhabdomyolysis (RM). Literatures were reviewed, which made us focus on the severe complications of DM.

The two adolescent patients were admitted urgently with T2DM complicated with ketoacidosis, whose chief complains were thirsty and polyuria for 6 days and 3 days respectively, drowsiness for 2 days and 2 hours. The skin was mottled and cold peripherally. The levels of blood glucose were 47.11mmol/L and 68.11mmol/L, corrected levels of serum sodium were 149.2mmol/L and 149.3 mmol/L, the osmolarity were 327.6 mOsm/L and 334.4mOsm/L. The urine ketone body were both positive, blood pH were 6.8 and 7.05. They were diagnosed as DKA and HHS. During the treatments of dehydration and insulin, the patients had oliguria because of poor liquid infusion. The blood creatine kinase (CK) of these patients elevated more than 10000IU/L with myoglobinuria charactered with black tea color urine. Based on above, RM were diagnosed. Acute renal failure occurred subseguently. We reviewed the literature of T2DM accompanied with DKA-HHS and RM. The mortality of HHS in children was rare. One report documented the mortality was 14% in children, which was similar to that of 15-20% approximately in adults. There was no document of case report or mortality of RM caused by T2DM with DKA-HHS. The literature documented RM usually caused by drugs, trauma and excessive muscular activity, etc. The normal saline infusion should be given at a rate of 1.5litres/hour at the beginning to maintain the urine output 200-300mL/hour in adults. The infusion of sodium bicarbonate is not necessary after effective liquid supplementation. Mannitol and loop diuretics may be useful. As for these patients, after the effective liquid infusion and anti-shock treatments, the urine ketone body turned to be negative and the urine color turned to be normal at the third day of admission. The CK level was decreased to normal at the 21-22th day. The two patients were all recovered finally.

The diagnosis of RM depends on the clinic manifestation and laboratory. T2DM with DKA-HHS and RM is very rare in pediatric patients. The main reason of RM of the two patients may be HHS and the inadequate liquid infusion. This kind of patients could be cured by adequate infusion. We are lack of the experience of the treatment to this kind of patients. So it is important for us to prevent the patients from RM, to recognize HHS earlier, to give enough liquid infusion and to prevent shock. These may be useful to decrease the mortality of RM and on the controry, to increase the survival rate of patients.

